# The duality of CXCR3 in glioblastoma: unveiling autocrine and paracrine mechanisms for novel therapeutic approaches

**DOI:** 10.1038/s41419-023-06354-2

**Published:** 2023-12-16

**Authors:** Travis Yui Hei Chan, Jenny Sum Yee Wong, Karrie Mei-Yee Kiang, Cherry Won Yuet Sun, Gilberto Ka-Kit Leung

**Affiliations:** 1 Division of Neurosurgery, Department of Surgery, School of Clinical Medicine, LKS Faculty of Medicine, The University of Hong Kong, Hong Kong, China; 2 Division of Vascular Surgery, Department of Surgery, School of Clinical Medicine, LKS Faculty of Medicine, The University of Hong Kong, Hong Kong, China; 3 Royal College of Surgeons in Ireland, University of Medicine and Health Sciences, Dublin, Republic of Ireland

**Keywords:** CNS cancer

## Abstract

Glioblastoma (GBM) is a highly aggressive brain tumor associated with limited therapeutic options and a poor prognosis. CXCR3, a chemokine receptor, serves dual autocrine–paracrine functions in cancer. Despite gaps in our understanding of the functional role of the CXCR3 receptor in GBM, it has been shown to hold promise as a therapeutic target for the treatment of GBM. Existing clinical therapeutics and vaccines targeting CXCR3 ligand expression associated with the CXCR3 axes have also shown anti-tumorigenic effects in GBM. This review summarizes existing evidence on the oncogenic function of CXCR3 and its ligands CXCL9, CXCL10, and CXCL11, in GBM, and examines the controversies concerning the immunomodulatory functions of the CXCR3 receptor, including immune T cell recruitment, polarization, and positioning. The mechanisms underlying monotherpies and combination therapies targeting the CXCR3 pathways are discussed. A better understanding of the CXCR3 axes may lead to the development of strategies for overcoming the limitations of existing immunotherapies for GBM.

## Facts

- The CXCR3 receptor and its ligands act through multiple signaling pathways in an autocrine and paracrine manner in glioblastoma (GBM) cells.
- Elucidating differences between the CXCR3-A and CXCR3-B pathways reveals opportunities for isoform-selective strategies. Current evidence suggests that selectively targeting CXCR3-A may suppress tumor growth while preserving CXCR3-B’s anti-tumor effects.
- Direct, indirect therapies and combination therapies exploiting the CXCR3 axes have shown to limit GBM growth, proliferation and migration in preclinical settings.

## Open Questions

- Which of the suggested CXCR3 immunomodulatory functions (immune cell recruitment, T cell polarization, or T cell positioning) is the most important for limiting GBM progression?
- Can we harness the CXCR3 axes to address the limitations of existing immunotherapies for GBM?

## Introduction

Glioblastoma (GBM), isocitrate dehydrogenase (IDH)–wildtype, is a WHO grade 4 malignant tumor [1] that accounts for 80% of primary brain neoplasms in adults [2]. The IDH gene mutation status of the tumor is critical for prognosis, with IDH-mutant GBM patients showing longer survival times and better treatment outcomes than IDH–wildtype GBM patients [3, 4]. The Stupp Protocol, the mainstay of GBM treatment since 2005, involves surgical resection, followed by concurrent chemoradiation and adjuvant temozolomide. However, the median survival is only 14.6 months, and the 2-year survival rate is 26.5% [5]. One therapeutic option that holds promise for GBM is immunotherapy. However, the immunosuppressive and immune-privileged environment of the brain has been shown to limit the efficacy of immune checkpoint inhibitors (ICIs) and chimeric antigen receptor (CAR) T-cell therapy in a clinical setting [6]. Thus, a better understanding of the tumor immune microenvironment is critical. Current research findings have demonstrated the importance of chemokine signaling in both tumor growth and immune cell functions, pathways that could be targeted to improve current immunotherapeutic options [7].

The C-X-C motif chemokine receptor 3 (CXCR3) is a chemokine receptor of the CXCR subfamily. It is a G protein–coupled receptor predominantly expressed on the cell surfaces of activated T cell, other leukocytic subtypes (dendritic cells, innate lymphoid cells, and natural killer [NK] cells), and non-immune related cells (astrocytes and fibroblasts) [8]. In cancer, the function of the CXCR3 receptor can be attributed to interactions between three pathways: the autocrine (intratumoral), paracrine (immune), and endothelial pathways. The autocrine pathway involves the tumoral secretion of chemokine ligands that bind to self-expressing CXCR3 receptors. The paracrine pathway is associated with CXCR3^+^ T cell recruitment and polarization [9–11]. The endothelial pathway mediates the angiostatic effects in cancer when CXCR3 receptors expressed on microvascular endothelial cells are activated [12–14]. However, the role of the endothelial pathway in GBM is underexplored, and thus is not covered in this review.

The clinical outcomes of tumors with differential CXCR3 expression have been disputed. Some studies have found that elevated CXCR3 expression is associated with a better prognosis in clear cell renal cell carcinoma [15, 16] and gastric cancer [17, 18]. Conversely, in cancers such as melanoma [19], breast cancer [20, 21], and GBM [22], the CXCR3 receptor has consistently been shown to be a poor prognostic factor. The paucity of studies on CXCR3 limits our understanding of the cross-talk between its autocrine and paracrine pathways, which are thoroughly discussed in this article. This review also describes the structural complexity of the CXCR3 receptor, which allows its gene to be translated into three distinct isoforms (CXCR3-A, CXCR3-B, and CXCR3-alt) expressed heterogeneously in cancer [23], and its four main ELR-negative chemokines (CXCL9, CXCL10, CXCL11, and CXCL4), which bind differentially to CXCR3 receptors, eliciting different downstream signalling mechanisms [8]. Therefore, treatments targeting the autocrine or paracrine pathways should take into account their potential interactions in the tumor microenvironment rather than focusing exclusively on the effect of the CXCR3 receptor on tumor cells. This review summarizes the scientific rationale and translational implications of GBM treatments that alter the CXCR3 associated pathways.

## Expression, localization, and function of CXCR3 in GBM cells

Each cancer’s proliferative and metastatic abilities depend on the expression levels of different CXCR3 isoforms in tumor cells. Several studies have shown that CXCR3 upregulation in vitro correlates with increased tumor proliferation, angiogenesis, and invasion in GBM [24, 25]. A similar trend has been observed in a clinical setting, suggesting that CXCR3 expression in GBM tissue is higher than in lower-grade astrocytoma tissue [22]. Few studies on CXCR3 have distinguished between the two major isoforms of the CXCR3 receptor (CXCR3-A and CXCR3-B) or investigated CXCR3-alt. One in vitro study demonstrated that CXCR3-A receptors present in all five GBM cell lines (i.e., U87, U118, U128, T98G, and A172) contributed to increased proliferation, while CXCR3-B receptors were expressed only in two of those cell lines (U118 and U128) and at considerably lower levels than CXCR3-A [25]. The regional localization of CXCR3-A is also discovered to be regulated by LRP1, a type 1 transmembrane protein involved in receptor-mediated endocytosis [26]. LRP1 is often upregulated in angiogenic areas, resulting in CXCR3 receptor internalization. This reduces the expression of the CXCR3 receptor on the cell surface, thereby abrogating angiogenesis. In contrast, LRP1 is downregulated in areas of tumor invasion, thus enabling upregulation CXCR3-A upregulation on cell membranes, causing tumor strand migration and promoting invasion. The regional discrepancies in CXCR3-A function suggests that it generally contributes to a proliferative phenotype in GBM, with different oncogenic phenotypes depending on its localization. Unlike CXCR3-A, the functional role of CXCR3-B in GBM has yet to be investigated due to its low expression profile in contrast with CXCR3-A. However, in cells of other cancers, such as renal cell carcinoma and breast cancer, CXCR3-B is known to cause apoptosis and angiostasis [27, 28].

The main challenge in differentiating between the two main CXCR3 isoforms is that in murine GBM models, only one isoform is expressed, with functions similar to those of CXCR3-A in humans [29]. Understanding the expression profiles of the two isoforms is critical, as a higher CXCR3-A: CXCR3-B ratio contributes to a higher risk of cancer metastasis compared to a lower CXCR3-A: CXCR3-B ratio [30]. Whether the same occurs in GBM cells has not been fully investigated. Regarding the functional role of CXCR3 ligands, elevated CXCL4, CXCL9, CXCL10, and CXCL11 levels have been clinically associated with a worsening prognosis [22, 31–33]. However, only the functional roles of intratumoral CXCL9 and CXCL10 have been investigated phenotypically [25, 31], with findings indicating that they contribute to cancer proliferation and migration. The available evidence suggests that the CXCR3-B isoform is characterized by lower expression and limited function compared to the CXCR3-A isoform in GBM. Table 1 summarizes the cancer-associated phenotypes of the CXCR3 isoforms and their ligands.

**Table 1:** Intratumoral expression of CXCR3 and its ligands in GBM.

Isoforms and ligands of CXCR3 in GBM	In vitro	In vivo	Clinical samples	Net effect
CXCR3 (overall expression, no differentiation between isoforms)	↑ [24–26, 77, 78]	N/A	↑ [22]	Increases tumor infiltration and angiogenesis [24, 25] and glioma cell invasion [24]
CXCR3-A	↑ [25, 26]	↑ [26]	N/A	Increase proliferation of glioma cells [30] Increases migration, when receptor expressed on cell membrane surface [26] Decreases angiogenesis upon internalization of the receptor [26]
CXCR3-B	↑ in a lesser amount than CXCR3A [25, 26]	N/A	N/A	Increases apoptosis and suppression of tumor growth (not confirmed in GBM) [27, 28]
CXCL4	N/A	N/A	↑ [79]	N/A
CXCL9 (Mig)	↑ [31]	↑ [25]	↑ [31, 33]	Increases proliferation of GBM cells [25, 31] and migration [31]
CXCL10 (IP-10)	↑ [25, 77]	↑ [25]	↑ [22]	Increases proliferation of GBM cells [25]
CXCL11 (ITAC)	N/A	Not expressed by C57BL/6 mice [80]	↑ [32]	N/A

The ↑ arrow indicates the increased expression of the respective CXCR3 receptor or the increased secretion of the respective ligand. *(The alternative names for CXCL9 is Mig, CXCL10 is IP-10, CXCL11 is ITAC)*.

## The autocrine pathway of CXCR3

**Fig. 1:**
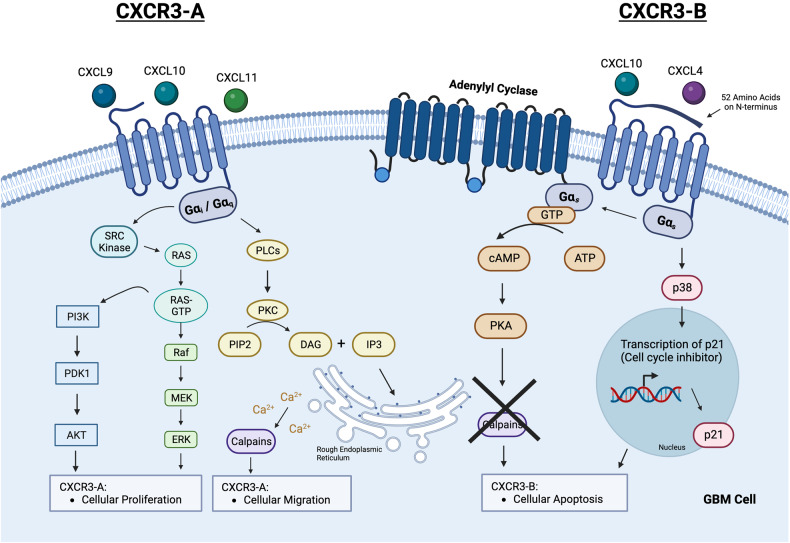
The downstream autocrine mechanisms of CXCR3-A and CXCR3-B in GBM. CXCL9, CXCL10, and CXCL11 bind to the CXCR3-A receptor. CXCR3-A couples with Gαi/Gαq. The activation of Gαi induces the activation of the SRC-RAS-ERK and PI3K/AKT downstream signaling pathways, leading to cellular proliferation. The activation of Gαq leads to phospholipase C-beta downstream signaling, inducing cell migration. CXCL10 and CXCL4 bind to the CXCR3-B receptor. CXCR3-B, which couples with Gαs, has a structural extension of 52 amino acids on its N-terminus. Upon chemokine binding, adenylyl cyclase and p38 are activated for their respective downstream signaling, inducing cellular apoptosis.

The CXCR3 receptor may undergo alternative splicing to produce three CXCR3 receptor isoforms: CXCR3-A, CXCR3-B, and CXCR3-alt. CXCR3-A receptors are structurally different from the CXCR3-B variants, with a lack of a 52 amino acids extension on the N-terminus [34], resulting in a shorter NH_2_ terminal extracellular domain in CXCR3-A compared to CXCR3-B. Such structural variation in CXCR3A drives the ligand specificities, allowing it to preferentially bind to CXCL9, CXCL10, and CXCL11, and couples with Gαi/q subunits [35]. Conversely, CXCR3-B preferentially binds to CXCL4 and CXCL10, and couples with Gαs subunit (Table 2). The structural and coupling differences between the CXCR3-A and CXCR3-B receptors lead to different downstream signaling pathways, which can result in contrasting cellular physiologies: CXCR3-A receptors are associated with cellular proliferation and invasion, while CXCR3-B receptors can trigger to cellular cancer cells apoptosis [30, 35] (Fig. 1).

**Table 2:** Summary of the structural, chemokine, and phenotypic differences between the CXCR3-A and CXCR3-B isoforms.

Structural, Chemokine and Phenotypic Differences	CXCR3 Isoforms	
	CXCR3-A	CXCR3-B
G-Protein-Coupled-Receptor Subunit	Gαi or Gαq [29]	Gαs [29]
Chemokines with a relative higher affinity	CXCL9, CXCL10, CXCL11 [29]	CXCL10, CXCL4 [29]
Overall Phenotype	Cellular proliferation and Cellular migration e.g. invasion, infiltration, metastasis [35, 37, 38]	Cellular apoptosis [39]

Regarding the proliferative function of CXCR3-A, the binding of CXCL9, CXCL10, and CXCL11 to the receptor activates Gαi, resulting in SRC kinase activation. This, in turn, activates the SRC–rat sarcoma–extracellular signal-regulated kinase (SRC-RAS-ERK) and phosphoinositide 3-kinase/protein kinase B (PI3K/AKT), and mitogen-activated protein kinase (MAPK) downstream signaling pathways, which are responsible for cell proliferation [36]. Regarding the invasive function of CXCR3-A, the same ligands binding to the receptor triggers Gαq instead of Gαi, leading to downstream phospholipase C-beta (PlCβ) signaling. This results in the cleavage of phosphatidylinositol 4,5-bisphosphate (PIP2) into diacylglycerol (DAG) and inositol-triphosphate (IP3). These cleaved products cause the endoplasmic reticulum to release calcium, activating the calpain protease, which is responsible for cell mobility and increased cell migration in cancer cells [37, 38].

On the other hand, CXCR3-B couples with Gαs. CXCL4 and CXCL10 binding to the CXCR3-B receptor activates adenylyl cyclase. This leads to the upregulation of intracellular cyclic adenosine monophosphate (cAMP) levels, which, in turn, inhibits calpain activation, thereby triggering apoptosis. An alternative downstream pathway to cellular apoptosis is the activation of p38, which increases the transcription of p21, thereby producing anti-proliferative effects [35]. In GBM, available evidence appears to favor the role of CXCR3-A receptor as the predominant CXCR3 isoform, whereas CXCR3-B is often downregulated in glioma cells in order to maintain a higher CXCR3-A: CXCR3-B ratio for tumor growth [25].

Notably, the known downstream pathways of CXCR3 in cancer significantly impact major protein kinases, such as AKT and PKA. However, targeting these protein kinases can be challenging as they are the central components of many cellular signaling pathways, and their inhibition may have unwanted side effects on healthy cells [39]. Therefore, focusing research on alternative downstream pathways of CXCR3 in cancer may lead to the development of more selective therapies with lower risks of off-target toxicity than those associated with broad kinase inhibitors. For instance, in steatohepatitis, the CXCR3 receptor has been found to be associated with mitochondrial function perturbation [40], while in macrophages, it has been shown to influence lysosomal trafficking [41]). Therapies aimed at reversing CXCR3-linked mitochondrial perturbations in diseases such as steatohepatitis could preserve general metabolic functions while blocking disease mechanisms. Similarly, modulating the impacts of CXCR3 on lysosomal activity in immune cells such as macrophages may help regulate inflammatory responses without broadly suppressing cellular signaling.

## The paracrine pathway of CXCR3

Besides their activity in tumor cells, CXCR3 receptors are also expressed heterogeneously on the cell membranes of immune cells, including T cells, dendritic cells, and NK cells [42]. Through paracrine regulation, the CXCR3 ligands CXCL9, CXCL10, and CXCL11 are mainly secreted by endothelial cells, fibroblasts, and some immune cells [11, 43]. In activated T lymphocytes, CXCR3-A isoform is the main upregulated isoform, but other isoforms of the CXCR3 receptor are also slightly elevated [44]. However, most studies on CXCR3 and immune cells have failed to specify the studied isoforms. We believe that this distinction is critical for future research because, as in the case of tumor cells, each CXCR3 isoform might activate different downstream pathways with varied effects.

As in other cancers, the CXCR3 paracrine pathway has been phenotypically demonstrated to have an overall anti-tumoral effect in GBM. An in vivo study compared the survival of tumor-bearing CXCR3-deficient and wild-type mice in an orthotopic GBM model, and found that the mice in the CXCR3-deficient group showed reduced recruitment of NK and natural killer T (NKT) cells and poorer median survival time [25]. However, the authors speculated that this phenomenon was likely due to a homeostatic defect rather than to a CXCR3-dependent effect [25], although no changes in the numbers of tumor-infiltrating CD4^+^ and CD8^+^ cells in the tumor was observed. This is surprising, as these two immune cell populations are commonly associated with CXCR3 and its ligands, and one would expect the increased recruitment of these immune cells to the tumor site. Indeed, another study reported higher infiltrative CXCR3^+^ CD4^+^ (T effector memory and T regulatory) and CD8^+^ (T effector memory only) expression in tumor samples from GBM patients than in blood samples from the same patients, although it remains unclear whether CXCR3 ligands were responsible for the direct recruitment of CXCR3^+^ immune cells [45]. The three potential mechanistic downstream paracrine pathways in cancer are further discussed in the following sections.

## Potential effects of CXCR3 on immune cells

### Immune cell recruitment

Studies have shown that CXCL9 and CXCL10 can directly recruit CD4^+^ and CD8^+^ T cells, and NK cells to the tumor site in various cancers [30, 46, 47]. Recently, *Brown et al*. demonstrated that the blockage of CXCR3 signaling in murine gliomas reduced the recruitment and stemness of tumor-infiltrating lymphocytes (TIL) in vivo [48]. However, a direct mechanistic explanation for this immune cell recruitment is lacking. One hypothesis could be related to interleukin-2 (IL-2), which in the pretreatment of CAR T cells has been shown to stimulate their proliferation and induce CXCR3 expression on their cell membrane surfaces through phosphatidylinositol 3-kinase (PI3K) activation, increasing the number of binding sites for CXCR3 ligands [49]. The fact that the PI3K pathway is activated universally in T cells in response to IL-2 involvement may explain why IL-2 secreted by CD4^+^ T cells at the tumor site induces CXCR3 expression in various T- cell subsets [50]. The inducement of CXCR3 expression on IL-2-activated T cells allows CXCR3 receptor binding by CXCL10, thereby increasing cytosolic Ca^2+^, and resulting in T cell recruitment and migration [51]. This mechanism provides a potential explanation for the well-established recruitment of CD8^+^ T cells to tumor sites [46, 47, 52]. The exact intracellular switch leading to Ca^2+^ increase is unknown, but it is possibly mediated by the phospholipase C (PLC) and phosphatidylinositol-kinase pathways. Other potential downstream candidates include calmodulin kinase, myosin light chain kinase, rho kinase, and other small guanosine triphosphates [53].

### T cell polarization

**Fig. 2:**
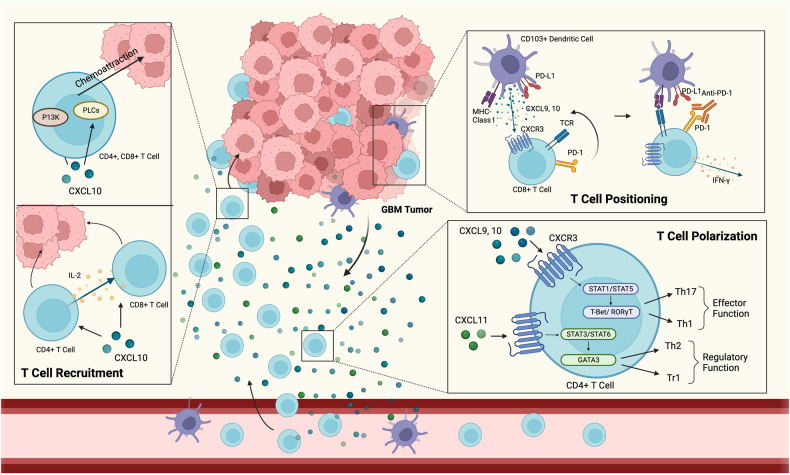
The paracrine mechanisms of CXCR3 for T cell recruitment, polarization, and positioning. **1**) T cell recruitment: CXCL9 and CXCL10 act as chemoattractants, recruiting CXCR3^+^ CD4^+^ and CD8^+^ T cells to the tumor site. **2**) T cell polarization: CXCL9 and CXCL10 induces CD4^+^ T cells differentiation into Th17 and Th1 cells, whereas CXCL11 binding to the CXCR3 receptor expressed on the cell surface membrane of CD4^+^ T cells induces their polarization into Th2 and Tr1 cells. **3**) T cell positioning: CXCL9 and CXCL10 binding to CXCR3^+^ T cells correct the alignment of CD8^+^ T cells with dendritic cells, improving the efficacy of anti-PD-1 therapy.

It is well known that T cells polarize into different effector cell types in response to the binding of different cytokines to their respective chemokine receptors in cancer [54]. The CXCR3 receptor is typically expressed at insignificant levels in naïve T cells. CXCL9 and CXCL10 binding to CXCR3^+^ naïve CD4^+^ T cells cause them to differentiate into Type 1T helper (Th1) and T helper 17 (Th17) cells by phosphorylating signal transducer and activator of transcription (STAT) 1, STAT 4, STAT 5, and TBX21/ retinoic acid related orphan receptor gamma t (T-Bet/ RORgammaT). On the other hand, CXCL11 binding to CXCR3^+^ naïve CD4^+^ T cells causes them to polarize to Type 1 regulatory T cells (Tr1) (IL-10 producing regulatory T cells) and Type 2 helper T (Th2) cells through the phosphorylation of STAT 3 and STAT 6 [8] or the activation of p70 kinase/ mTOR pathways [54] or the GATA-binding protein 3 pathways. This phenomenon, whereby different ligands bind to the same CXCR3 receptor but trigger downstream pathways, is commonly known as ligand bias signaling (Fig. 2).

### T cell positioning

A recent landmark study by Chow et al. found no significant changes in absolute CD8^+^ effector cells in C57BL/6 mice compared to CXCR3-deficient mice in a melanoma model, suggesting that, contrary to previous findings, the CXCR3 receptor is not involved in T cell infiltration. This, in turn, suggests that in a therapeutic setting, increased levels of CXCL9 and CXCL10 secreted by CD103^+^ dendritic cells binding to CXCR3^+^ T cells would enable the correct alignment of T cells with dendritic cells. This alignment would enable CXCR3^+^ T cells to receive anti-PD-1 (antitumor) immunotherapeutic signals, thus improving the efficacy of this treatment [55] (Fig. 2). The importance of CXCL9 and CXCL10 in anti-PD-1 therapy has also been supported by other studies, but rather than dendritic cells, macrophages (and, to a lesser extent CD11b^+^ myeloid cells) [56] have been suggested to be the predominant source of these ligands after PD-1 blockade [43].

The complex interplay between the CXCR3 receptor and its ligands in GBM in an immune context requires further mechanistic elucidation. Although it contradicts previous findings, the recent discovery of CXCR3-dependent T-cell alignment with dendritic cells upon anti-PD-1 treatment indicates an exciting new direction. Future studies should focus on identifying the predominant immune cells responsible for CXCL9 and CXCL10 secretion in GBM and determining whether similar T-cell positioning and realignment occur with different immunotherapies. A deeper understanding of the CXCR3 axes may further unveil new therapeutic targets to improve T-cell infiltration and reactivation in GBM.

## Therapeutic implications

The role of the interaction between CXCR3 receptors and the chemokines in GBM progression provides the possibility of using CXCR3 modulators as a targeted therapy. Pre-clinical research on mouse models supports the potential of targeting chemokine signaling and receptors for immunotherapy, chemotherapy, and antiangiogenic combination therapy [57].

### Direct monotherapy

**Fig. 3:**
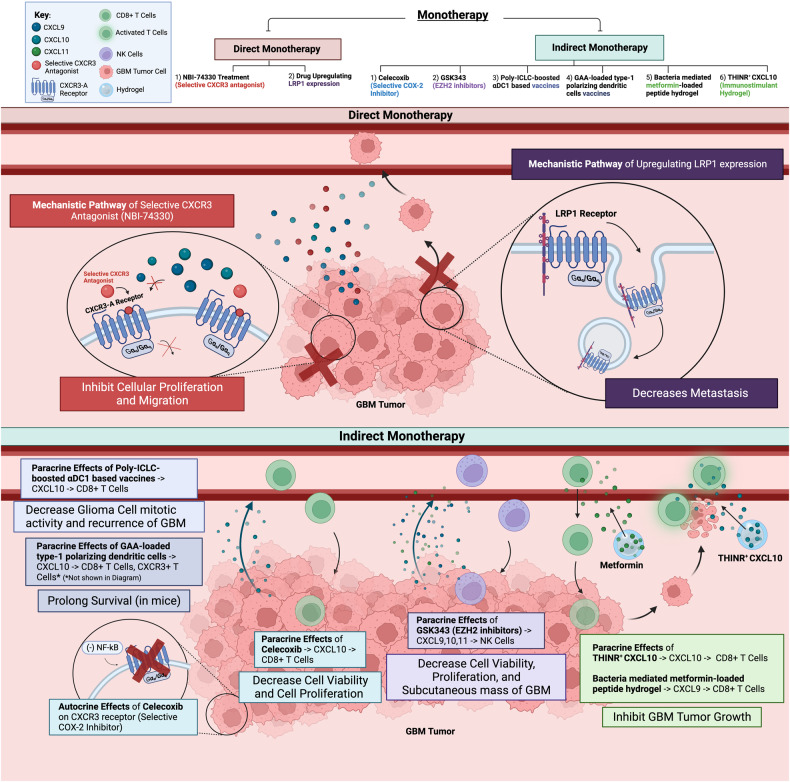
Direct and Indirect Monotherapies’ Mechanistic Pathways. Direct monotherapies: **1**) A selective CXCR3 antagonist binds to the CXCR3-A receptor in the tumor cells, inhibiting CXCL9, CXCL10, and CXCL11 binding to the receptor and downstream signaling for cell proliferation and migration. **2**) Drugs upregulating LRP1 expression cause the internalization of the CXCR3-A receptor, restricting GBM metastasis. Indirect monotherapies: **3**) Celecoxib inhibits the NF-kB signaling pathway, reducing CXCR3 expression. Celecoxib also increases CXCL10 expression, promoting T cell recruitment. **4**) GSK343 (EZH2 inhibitor) increases CXCL9, CXCL10, and CXCL11 expression, promoting NK cell recruitment. **5**) Both Poly-ICLC boosted αDC1 based vaccines and GAA-loaded type 1 polarizing dendritic cell vaccines increase the expression of CXCL10, promoting T cell recruitment. **6**) A bacterially mediated metformin-loaded peptide hydrogel and THINR^+^ CXCL10 upregulate the CXCL9 and CXCL10, respectively, promoting for T cell recruitment.

Research has revealed that GBM growth is primarily regulated through the CXCR3-A receptor in the autocrine signaling pathway. Targeting this axis directly with monotherapies to block the CXCL9/CXCL10/CXCL11–CXCR3 axis or to regulate CXCR3-A expression on the tumor cell membrane surface holds promise. The selective CXCR3 inhibitor NBI-74330 has been shown to reduce proliferation in vitro and to improve the survival of both wildtype and CXCR3-deficient mice by preventing ligand binding to the CXCR3 receptor in vivo [25]. However, this inhibitor does not increase lymphocyte migration, indicating that CXCR3 may not be involved in T cell trafficking. More specific inhibitors, such as SCH546738, which inhibits CXCR3-A but not the anti-tumoral CXCR3-B isoform, may have a more potent anti-tumor effect, since evidence suggests that it curbs tumor growth in GBM models while preserving the effects of CXCR3-B [23] (Fig. 3, Table 3). Future research should focus on the development of drugs that specifically inhibit the tumor-promoting effects of CXCR3-A receptors while still maintaining the anti-tumoral effects of CXCR3-B receptors. Taken together, the current evidence strongly suggests that the targeted selective inhibition of CXCR3-A receptors can prevent GBM growth.

The mechanism by which LRP1 regulates CXCR3-A was discussed earlier. CXCR3-A binds to the alpha chain of LRP1, leading to CXCR3-A receptor internalization through a claritin-dependent pathway. The internalization of CXCR3-A reduces the membrane expression of CXCR3-A on cell membranes, limiting the tumor’s ability to survive. A pre-clinical study using the stable receptor-associated protein (RAP), an LRP1 inhibitor, observed increased glioma cell migration [26]. This is because the inhibition of LRP1 receptor–CXCR3-A binding leads to reduced CXCR3-A internalization, which supports our hypothesis that upregulation of LRP1 in the tumor may have the opposite effect, promoting CXCR3-A endocytosis and reducing GBM invasion (Fig. 3). The development of LRP1-promoting drugs represents a potential new strategy for preventing CXCR3-A-mediated metastasis.

### Indirect monotherapy

Besides targeting the CXCR3 receptor directly, modulating other upstream or downstream pathways of the CXCR3 axes can affect the secretion of the CXCR3 ligands and sequentially affect the activation of the autocrine and paracrine CXCR3 axes. Indirect monotherapies target these pathways. They involve increasing ligand secretion through well-known upstream signaling pathways that regulate the production of CXCR3 ligands, such as the nuclear factor kappa-light-chain-enhancer of activated B cells (NF-kB) and histone methyltransferase enhancer of zeste homolog 2 (EZH2) pathways, within tumor cells in an autocrine manner. Dendritic cell vaccines and nanomaterial hydrogels are other targeted therapies used to augment the immune functions of CXCR3.

Therapeutic inhibitors of the NF-kB signaling pathway may affect the expression of the CXCR3 receptor and its ligands to suppress tumorigenesis. One study found that celecoxib, a cyclooxygenase (COX-2) inhibitor, induced apoptosis through inhibitory signaling on the Akt activation pathway in low-grade gliomas in a dose-dependent manner [58]. While this study did not investigate the role of CXCR3 in regulating the Akt pathway, it showed that celecoxib inhibited the NF-kB signaling cascade. It has been well documented that blocking NF-kB decreases CXCR3 expression and CXCL10 in an autocrine feedback loop, thereby reducing glioblastoma cell proliferation [59]. However, in a paracrine manner, another study demonstrated that celecoxib enhanced overall CXCL10 expression in the glioma microenvironment through a decrease in myeloid-derived suppressor cells (MDSC), thus upregulating infiltration of CD8^+^ T cells into GBM [60] (Fig. 3). Therefore, COX-2 inhibitors may have therapeutic benefits for GBM patients, as they may suppress glioma progression in both an autocrine and paracrine manner.

The EZH2 GSK343 inhibitor represents another potential therapy for eliciting CXCR3-associated immune responses in GBM. In vitro, this inhibitor has been shown to cause glioma cell apoptosis by increasing the expression of apoptotic protein expression, including Bax, p53, and caspase-9. Although its anti-proliferative effects may not result from the direct inhibition of the CXCR3 receptor and ligand alterations, the GSK343 inhibitor has been suggested to increase the expression of CXCL9, CXCL10, and CXCL11 in the GBM environment, which enhances NK cell infiltration, thereby inhibiting GBM tumor growth. The GSK343 inhibitors have been shown to reduce glioma cell viability, decrease subcutaneous GBM mass, and exert anti-proliferative effects in vitro, in vivo, and ex vivo, respectively [61] (Fig. 3, Table 3). This suggests that targeting the upstream secretion of CXCL9, CXCL10, and CXCL11 ligands could be a strategy to modulate CXCR3 function.

From a translational perspective, phase I and II clinical trials of dendritic cell vaccines (polyinosinic-polycytidylic acid and carboxymethylcellulose (poly-ICLC) boosted αDC1) have shown promise for reducing GBM recurrence through a CXCR3-dependent mechanism. In one trial, the upregulation of CXCL10 and interferon-α caused by such a vaccine promoted the trafficking of CD8^+^ T cells and CD68^+^ macrophages into the GBM site, thereby reducing active mitotic activity in the tumor [62]. Among 19 of the 22 patients with recurrent GBM who received four doses, 58% showed increased response of chemokines, including interferon gamma (IFN-γ), interferon alfa (IFN-α), and CXCL10, leading to sustained immune responses and prolonged patient survival. Moreover, 41% of the 22 patients were progression free for more than one year, and 23% remained progression free after the end of the trials [62]. Similar results were obtained from an in vivo study of a glioma-associated antigen-loaded type-1 polarizing dendritic cell vaccine. This study also observed a positive feedback loop of high CXCL10 production. CXCL10 activated CXCR3^+^ effector T cells, which stimulated T cells to release more CXCL10, further enhancing the chemoattraction of CXCR3^+^ T lymphocytes and antigen-specific cytotoxic T lymphocytes to gliomas [63] (Fig. 3). This evidence supports the long-term efficacy of the vaccine, rather than a transient effect [64]. A recent phase I/II trial testing the combined effects of poly-ICLC and multipeptide IMA950 vaccines on GBM patients reported a longer overall median survival of 19 months. Ongoing phase I/II trial testing the effects of combinations of IMA950/poly-ICLC vaccines with existing immunotherapies, such as varlilumab and pembrolizumab on recurrent GBM aim to identify the optimal T cell response against GBM [64]. Phase III and IV clinical trials may confirm the efficacy of vaccines targeting the CXCR3 axes in combination with immunotherapies.

A novel therapeutic strategy is the use of nanomaterials, such as hydrogels. In one study, bacterially-mediated metformin was loaded into peptide hydrogel to produce Melittin-RADA32-Metformin (MRM) coated spores. This nanodrug triggered immune responses against GBM through increased CXCL9 expression in the tumor environment. This increased the infiltration of CD8^+^ T cells (including CXCR3^+^ CD8^+^ T cells), which inhibited GL261 glioma cell growth [65] (Fig. 3). A similar study employed a tumor-homing immune nanoregular (THINR) for the local release of CXCL10 in residual GBM tumors to exert tumoricidal effects. The local stimulation of CXCL10 allowed the precise recruitment of activated CD8^+^ T cells to induce the apoptosis of metastasizing glioma cells in vitro (GL261 cell line) and in vivo. The survival of mice treated with THINR^+^ CXCL10 was prolonged for four additional months, compared to the control group treated with PBS alone [66] (Fig. 3).

In summary, celecoxib affects the expression of the CXCR3 receptor and its ligands in both autocrine and paracrine manners to suppress tumorigenesis. It reduces CXCR3 receptor and CXCL10 expression on the tumor cells while increasing CXCL10 expression in the glioma microenvironment. GSK343 inhibitor induces glioma cell apoptosis and increases CXCL9, CXCL10, and CXCL11 expression in the GBM environment. Dendritic cell vaccines restrict GBM recurrence by upregulating CXCL10 and IFN-α to promote CD8^+^ T cells and CD68^+^ macrophage recruitment to the GBM site. Finally, the local release of CXCL10 by nanomaterial hydrogels recruits activated CD8^+^ T cells and induces the apoptosis of metastasizing glioma cells. Thus, current evidence suggests that targeting the upstream secretion of CXCR3 ligands may be an effective therapeutic strategy for GBM (Table 3).

### Combination treatment with immunotherapy

ICIs and CAR T cells show promise for GBM regression and T cell proliferation and activation [67]. However, these therapies present translational challenges. One limitation of the ICI PD-L1 is the low infiltration of tumor-infiltrating lymphocytes into GBM [68]. CAR T cell immunotherapy for GBM faces a similar obstacle due to the physical barrier of the perivascular areas in solid tumors, which restricts intratumoral CAR T cell infiltration [69]. Hence, the efficacy of immunotherapy for GBM as a standalone treatment remains suboptimal. However, accumulating pre-clinical evidence suggests the potential to overcome these limitations and improve the efficacy of the immunotherapy through CXCR3 ligand–targeted therapies, including CXCR3 ligand–modulating protein inhibitors, immuno-virotherapy, and fusion proteins.

**Fig. 4:**
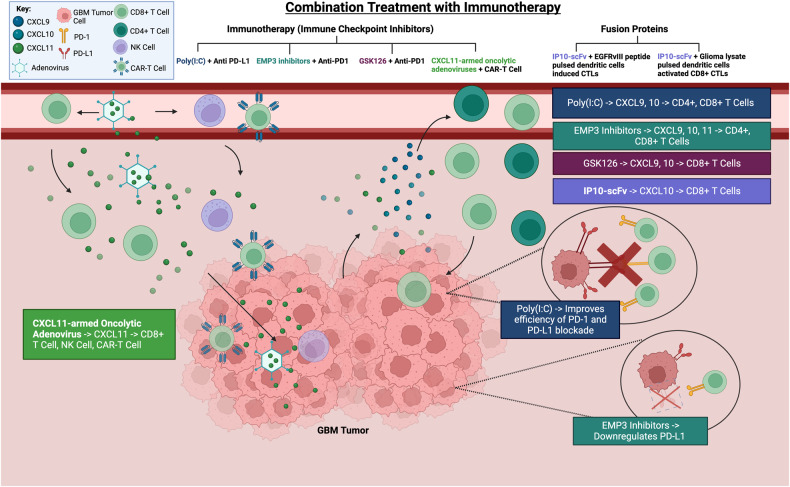
Combination Treatment with Immunotherapy’s Mechanistic Pathways. **1**) Poly(I:C) combined with anti-PD-L1 therapy increases CXCL9 and CXCL10 secretion, promoting T cell recruitment. **2**) An EMP3 inhibitor combined with anti-PD-1 increases CXCL9, CXCL10, and CXCL11 secretion, promoting T cell recruitment. **3**) GSK126 (EZH2 inhibitor) combined with anti-PD-1 increases CXCL9 and CXCL10 secretion, promoting T cell proliferation and recruitment. **4**) CXCL11-armed oncolytic adenoviruses with CAR T cell therapy increased CXCL11 secretion, promoting T cell, NK cell and CAR T cell infiltration. **5**) IP10–scFv fusion protein therapies increase CXCL10 secretion, promoting T cell recruitment.

Polyinosinic-polycytidylic acid, or poly(I:C), improves anti-PD-L1 efficacy by increasing CXCL9 and CXCL10 secretion in the paracrine pathway. This promotes the infiltration of CXCR3^+^ CD8^+^ and CD4^+^ T cells into GBM [70] (Fig. 4). One study investigated the immunomodulatory effects of epithelial membrane protein 3 (EMP3) on GBM growth. EMP3 knockout combined with anti-PD-1 therapy resulted in tumor growth inhibition by promoting T cell (CD4^+^ and CD8^+^) infiltration mediated by the CXCR3 axes. EMP3 inhibition further elevated IFN-γ, which induces CXCR3 ligand production contributing to the increased production of cytotoxic immune cells [71] (Fig. 4). Another study found that the EZH2 inhibitor GSK126 combined with anti-PD-1 treatment significantly boosted immune infiltration by increasing CXCR3 ligand expression. GSK126 played a vital role in promoting IFN-γ, which increased CXCL9 and CXCL10 expression in the tumor, promoting T cell proliferation, maturation, activation, and chemotaxis into GBM, thereby inhibiting tumor growth and prolonging survival. The ability of GSK126 to permeate the blood–brain barrier also improved the ability of anti-PD-1 antibodies to cross the barrier, thus enhancing therapeutic efficiency [72] (Fig. 4) (Table 3). Given the preclinical evidence suggesting the combined use of CXCR3 ligand–targeted therapies and ICIs promotes lymphocyte infiltration into GBM, the next step is to test their clinical applications.

**Table 3:** Summary of the overall effects of monotherapies and combination treatments for GBM.

Monotherapy				
	Pre-clinical (In vivo/ In vitro/ Ex Vivo) / Clinical Samples / Clinical trials	Effects of therapy on CXCR3 or ligands expression on tumor cells (Altering **Autocrine** functions)	Effect of therapy on CXCR3 or Ligands expression on immune cells (Altering **Paracrine** functions)	Consequences and Overall therapeutic effects on GBM
**Direct Monotherapy**				
**NBI-74330 Treatment (Selective CXCR3 antagonist)**	In vitro: Human glioma cell lines (A172, U118, T98G, U87, and U138) and Murine cell lines (GL261) [25] In vivo: Murine model of malignant glioma (WT C57BL/6) [25]	Competitively binds to CXCR3 compared to CXCL9, CXCL10 to induce inhibitory effects of CXCR3 [25]	No statistically significant effect on increase infiltrating microglia and lymphocytes in GBM [25]	Prolonged survival in vivo [25] Decrease GBM malignant potential [25]
**Drug upregulating LRP1 expression**	In vitro: Gallus (U87-CTRL, and U87-CXCR3-A) and Patient derived glioma cell lines [26] In vivo: Chicken embryo model (CAM) [26] Ex vivo: Analysis of CXCR3 and LRP1 expression in glioma samples from patients [26]	Increases internalization and endocytosis of CXCR3-A [26] Decrease in CXCR3-A receptor on glioma cell membrane [26]	N/A	Increase in LRP1 receptor expression reduces glioma invasiveness and metastasis [26] Decreases glioma cell proliferation migration and survival [26] LRP1 was strongly expressed in the less invasive part of the tumor cell, while CXCR3 was localized in the cytoplasm [26]
**Indirect Monotherapy**				
**Celecoxib (Selective COX-2 inhibitor)** [59, 60]	In vitro: Glioblastoma stem cells (GSC) [59], Glioma cell lines [60] In vivo: GSC-bearing glioma model (C57BL/6) [59], Murine model (WT C57BL/6) [60]	Inhibition of CXCR3 expression mechanistically through the inhibition of NF-kB signaling [60]	Increased expression of CXCL10 endogenously leads to increase in CD8^+^ T cell infiltration into GBM tumor environment [60]	Decreased glioma cell viability and cell proliferation [59] COX-2 blockade suppress glioma genesis [60]
**GSK343 (EZH2 inhibitors (Histone methyltransferase enhancer of zeste-homolog-2)**	In vitro: Human GBM cell lines (U87, U138MG, and A172) [61] In vivo: BALB/c nude female mice; Xenograft model of glioblastoma [61] Ex vivo*:* Patient-derived glioblastoma cells [61]	N/A	Increased CXCL9, CXCL10 and CXCL11 in regulating anti-tumor immune cell tracking e.g. NK cells for innate immune response [61]	Decreased cell viability (In vitro) [61] Decreased subcutaneous GBM mass (In vivo) [61] Anti-proliferation of glioma cells (Ex vivo) [61]
**Poly-ICLC-boosted αDC1 based vaccines**	Phase I/ II Clinical Trial [62]	N/A	Increased CXCL10 after first and fourth vaccine, causing increase CD8^+^ T cell trafficking and trafficking into GBM [62] Clinical Data: 58% of patients showed upregulated CXCL10 [62]	Inhibition of glioma cell mitotic activity and GBM tumoral growth [62] Reduction in recurrence of GBM [62] Clinical Data: 41% of patients were progression-free for more than one year; 23% of patients remained progression free after trials [62]
**Glioma associated antigen (GAA)-loaded type-1polarizaing dendritic cells vaccines**	In vitro: Murine cell line (CD40L^+^ J558 myeloma cells, TAP2−/−RMA-S thymoma cells, and GL261 glioma cells) [63] In vivo: Murine model (WT C57BL/6) [63]	N/A	Increased CXCL10 production to increase CD8^+^ T cell and CXCR3^+^ T cell infiltration into glioma [63]	Prolong survival in mice [63]
**Bacteria mediated metformin-loaded peptide hydrogel**	In vitro: Murine glioma cell lines (GL261, U87, and U251) [65] In vivo: Murine model (female C57BL/6) [65]	N/A	Increased CXCL9 in GBM tissue, leading to increased infiltration CXCR3^+^ cytotoxic CD8^+^ T cells [65]	Inhibit GBM tumor growth [65]
**THINR-CXCL10 (Immunostimulant Hydrogel)**	In vitro: Murine glioma cell line (GL261) [66] In vivo: Murine Intracranial GBM model (female C57BL/6J) [66] Clinical samples: Patient tumor sections [66]	N/A	Exogenous CXCL10 release through THINR hydrogel system recruited activated CD8^+^ T cells to induce cytotoxicity and apoptosis on the disseminated tumoral cells [66]	Prolonged survival for 4 months (In Vivo) [66] Inhibit GBM tumor growth [66]
**Combination Treatment**				
		Effect of therapy on CXCR3 or Ligands expression on immune cells (Altering **Paracrine** functions)	Overall therapeutic effects on the second therapy	Consequences and Overall therapeutic effects on GBM
**Combination Treatment + Immunotherapy**				
**Poly(I:C) (Polyinosinic-polycytidylic acid) + Anti-PD-L1 therapy (Immune checkpoint inhibitor)**	In vitro: Primary human glioblastoma cells (patient-derived tumor tissue samples) [70]	Increased secretion of CXCL9 and CXCL10, leading to increased infiltration and proliferation of CD8^+^ T cells and CD4^+^ T cells (CD8+ higher in proportion) [70] Downregulate CXCR3 on CD4^+^ and CD8^+^ T cells [70]	Increased Poly(I:C) improves the effect of PD-1/PD-L1 blockade [70]	Enhanced immune activation and response on GBM apoptosis and proliferation inhibition [70]
**EMP3 inhibitors (Epithelial Membrane Protein 3) + Anti-PD1 therapy (Immune checkpoint inhibitor)**	In vitro: Murine glioma cells (GL261, RAW 264.7, and BV-2) [71] In vivo: Murine Intracranial GBM model (female C57BL/6) [71] Clinical samples: GBM wild-type IDH1 tissue samples (27 patients) [71]	Increased IFN-γ induced secretion of CXCR3 ligands to increased infiltration of CD4^+^ and CD8^+^ [71]	EMP3 inhibition downregulates PD-L1 to improve anti-PD1 efficacy [71]	Knockout EMP3 improves efficacy of anti-PD1 therapeutic responses to improve GBM survival [71]
**GSK126 (EZH2 inhibitor) + Anti-PD1 therapy (Immune checkpoint inhibitor)**	In vitro: Human GBM cell lines (A127 and U251) and Murine cell lines (GL261 and CT2A) [72] In vivo: Murine model (female albino C57BL/6) [72]	Increase CXCL9 and CXCL10, leading to increase in CD8^+^ T cell proliferation, maturation, activation and chemoattraction into GBM [72]	GSK126 modulates epigenetic changes to reverse immunosuppressive-environment to improve anti-PD1 therapy [72]	Decreased tumor growth [72] Leads to prolonged survival [72]
**CXCL11-armed oncolytic adenoviruses** + **CAR T cell immunotherapy**	In vitro: Human cell lines (293 A, 293 T, A549, U251, A172, U87, T98G, H4) and Murine cell line (GL261) [74] In vivo: Murine model (female C57BL/6) [74] Clinical samples: Peripheral blood mononuclear cells of healthy donors and GBM patients; GBM tumor samples [74]	Increased exogenous CXCL11 (dominant ligand for CXCR3 expressed on T cells), leading to increase chemoattraction of activated CD8^+^ T cells and NK cells [74]	Increased CAR T cell infiltration into GBM tumor site, improving efficacy of CAR T cell immunotherapy [74]	Promote apoptosis of GBM tumor cell [74] Leads to prolonged survival [74]
**Fusion Proteins + Immunotherapy**				
**IP10-scFv (Fusion Protein)** + **EGFRvIII peptide pulsed dendritic cells induced CTLs**	In vitro: Human GBM cell line (U87), Human embryonic kidney 293 cell line (HEK-293), Mouse NIH 3T3 embryonic fibroblast cell line, human Hep3B hepatocellular carcinoma cell line [75] In vivo: Xenograft mice model (female BALB/c-nu) [75]	IP–scFV induces CXCL10 expression, leading to increased CD8^+^ T cells and CXCR3^+^ T cells [75]	Synergistic effect on T cell immunity against glioma cells [75]	Inhibits glioma growth [75] Leads to prolonged survival [75]
**IP10-EGFRvIIIscFv + Glioma lysate pulsed dendritic cells activated CD8** + **CTLs**	In vitro: Murine glioma cell line (GL261) and Human GBM-astrocytoma epithelial like cell line (U87) [76] In vivo: Murine model (C57BL/6) [76]	IP-scFV induces CXCL10 expression for recruitment of CXCR3 positive CD8^+^ T cells [76]	Synergistic effect on T cell immunity against glioma cells and induce apoptosis [76]	Inhibits glioma growth [76] Leads to prolonged survival [76]

The efficacy of CAR T cell immunotherapy is hindered by the migratory chemokine signaling mismatch in the tumor microenvironment (TME), which limits CAR T cell infiltration [69]. To overcome this limitation, one study tested a therapy based on oncolytic adenoviruses engineered with CXCL11 to reprogram the tumor microenvironment. This immuno-virotherapy had a dual effect. The oncolytic adenoviruses infected tumor cells and induced apoptosis [73], while CXCL11 acted as a chemoattractant for CAR T cells, activated T cells, and NK cells, promoting their infiltration into GBM. The result was prolonged patient survival [74] (Fig. 4). Similarly, stimulating CAR T cells with IL-2 and GM-CSF has been shown to induce CXCR3 expression on CAR T cells, leading to enhanced infiltration into hepatocellular carcinoma [49], suggesting that this strategy can lead to improvements in CAR T cells immunotherapy for GBM (Table 3).

The interferon gamma inducible protein 10-single chain variable fragment (IP10–scFv) fusion proteins, a combination of CXCL10 chemokine with a single chain variable fragment with epidermal growth factor receptor variant III (EGFRvIII), represents another strategy for enhancing immunotherapy. One study showed that IP10–scFv fusion protein and glioma-specific cytotoxic T lymphocyte combination treatment inhibited mouse tumor growth. Increased IP10–scFv production targeted EGFRvIII-expressing glioma cells, leading to an increased chemotactic attraction of CD8^+^ T lymphocytes and CXCR3^+^ T cells infiltrating into the tumor. It also served to prolong the residence time of cytotoxic T lymphocytes, enhancing cytotoxicity in the glioma [75]. A similar combination treatment of IP10–scFv fusion protein and dendritic cell–induced CD8^+^ cytotoxic T lymphocytes has been shown to recruit CXCR3^+^ CD8^+^ T cells, leading to cytotoxicity and apoptosis of glioma cells, thereby inhibiting glioma growth and prolonged survival [76] (Fig. 4) (Table 3).

In brief, all CXCR3 therapies aim to induce the secretion of CXCR3 ligands (CXCL9, CXCL10 and CXCL11) to promote T cell infiltration into GBM, thereby enhancing the effects of immunotherapies. Poly(I:C) combined with anti-PD-L1 increases the secretion of CXCL9 and CXCL10 to recruit CD4^+^ and CD8^+^ T cells, improving the effect of PD-1/PD-L1 blockade. CXCR3 ligand–modulating protein inhibitors, (EMP3 inhibitor and GSK126 [EZH2 inhibitor]) combined with anti-PD-1 leads to increased CXCR3 ligand secretion to recruit T cells to magnify the anti-PD-1 effects, thus arresting GBM growth and prolonging survival. CXCL11-armed oncolytic adenoviruses increase the secretion of CXCL11 to activate T cells and NK cells, thereby improving CAR T cell infiltration. Fusion protein therapies induce CXCL10 expression for T cell infiltration, inhibiting glioma growth and improving survival. These combination therapies hold promise for the treatment of GBM.

## Conclusion

Recent studies have demonstrated the dual pro-/antitumor of CXCR3 in various cancers, including in melanoma, breast cancer, and renal cell carcinoma [9, 52]. This is also the case with GBM, in which the upregulation of intratumoral CXCR3-A and the ligands CXCL9, CXCL10, and CXCL11 levels is associated with a poor patient prognosis. The role of the autocrine (tumor-promoting) downstream pathways of CXCR3 in GBM has been extensively studied. However, other pathways should be more thoroughly explored for further therapeutic interpretation. Moreover, the exact immune function of CXCR3 in tumors is controversial. While most preclinical studies on CXCR3 ligand–targeting therapeutic compounds indicate that CXCR3 ligand expression promotes the recruitment of CXCR3^+^ CD4^+^ and CD8^+^ T cells to the tumor site, other studies have suggested that this is not the case. Nevertheless, therapeutic interventions aimed at modulating CXCR3 ligand expression have succeeded both in vitro and in vivo. Besides recruitment, the potential functions of CXCR3 include T cell priming and positioning, both of which have been proven in other cancers. These findings provide strong support for the potential use of such therapeutic compounds in a clinical context. This translational process is exemplified by an ongoing phase I/II clinical trial on the combined use of poly-ICLC vaccines and immunotherapies for GBM. Further research is needed to optimize combination treatment strategies for effectively targeting the CXCR3 pathways. Moreover, compounds such as the selective CXCR3 inhibitor NBI-74330, CXCR3 ligand–releasing hydrogels, and fusion proteins should also be considered for clinical trials to advance the treatment of GBM.

### Reporting summary

Further information on research design is available in the Nature Research Reporting Summary linked to this article.

### Supplementary information

Reporting Summary

## Data Availability

Data sharing is not applicable, as no datasets were generated or analyzed during this study.
